# The Collagen Origin Influences the Degradation Kinetics of Guided Bone Regeneration Membranes

**DOI:** 10.3390/polym13173007

**Published:** 2021-09-05

**Authors:** Marta Vallecillo-Rivas, Manuel Toledano-Osorio, Cristina Vallecillo, Manuel Toledano, Raquel Osorio

**Affiliations:** 1 Faculty of Dentistry, Colegio Máximo de Cartuja s/n, University of Granada, 18071 Granada, Spain; mvallecillo@correo.ugr.es (M.V.-R.); cvallecillorivas@hotmail.com (C.V.); toledano@ugr.es (M.T.); rosorio@ugr.es (R.O.); 2 Medicina Clínica y Salud Pública PhD Programme, 18071 Granada, Spain

**Keywords:** bone regeneration, collagen membrane, hydrolytic degradation, collagen origin, bacterial collagenase, trypsin digestion, degradation testing, degradation kinetics

## Abstract

Collagen membranes are currently the most widely used membranes for guided bone regeneration; however, their rapid degradation kinetics means that the barrier function may not remain for enough time to permit tissue regeneration to happen. The origin of collagen may have an important effect on the resistance to degradation. The aim of this study was to investigate the biodegradation pattern of five collagen membranes from different origins: Biocollagen, Heart, Evolution X-fine, CopiOs and Parasorb Resodont. Membranes samples were submitted to different degradation tests: (1) hydrolytic degradation in phosphate buffer saline solution, (2) bacterial collagenase from *Clostridium histolyticum* solution, and (3) enzyme resistance using a 0.25% porcine trypsin solution. Immersion periods from 1 up to 50 days were performed. At each time point, thickness and weight measurements were performed with a digital caliper and an analytic microbalance, respectively. ANOVA and Student–Newman–Keuls tests were used for comparisons (*p* < 0.05). Differences between time-points within the same membranes and solutions were assessed by pair-wise comparisons (*p* < 0.001). The Evolution X-fine collagen membrane from porcine pericardium attained the highest resistance to all of the degradation tests. Biocollagen and Parasorb Resodont, both from equine origin, experienced the greatest degradation when immersed in PBS, trypsin and *C. histolyticum* during challenge tests. The bacterial collagenase solution was shown to be the most aggressive testing method.

## 1. Introduction

Nowadays, both in oral implantology and periodontology, the management and treatment of alveolar deficiencies are major clinical issues. Guided bone regeneration (GBR), a technique based on the use of membranes creating space to be filled with new bone, was originally hypothesized more than three decades ago [1,2]. This concept implies the exclusion of those cells types that proliferate faster than bone cells, such as those destined to form epithelial and connective tissue [3,4]. At the same time, space is maintained to allow cells capable of forming bone to grow [3]. These selective repopulation procedures are applied in regenerative periodontal clinical situations and to treat various intraoral bone defects, not limited to implant purposes [5].

For a material to be used as a membrane for GBR, some fundamental requirements must be fulfilled: biocompatibility, cell-occlusiveness, space maintenance, tissue integration and easy manipulation [1,6,7,8,9]. Volumetric stability of the membrane over time is also pivotal, in order to remain for long enough for regeneration to take place [10]. A membrane should be capable of being degraded or resorbed at the same rate that bone growth occurs [7]. It has been established that for periodontal regeneration, 4–6 weeks are necessary; however, for GBR procedures, a longer period of more than 6 months is recommended [1,11]. Membranes for GBR can be broadly divided into two main groups: resorbable and non-resorbable. Non-resorbable membranes remain stable over time without undergoing degradation processes. On the contrary, these membranes require a second surgery for their removal [8,12]. Due to this, resorbable membranes are used more frequently for GBR, alleviating the discomfort of the patient caused by a second surgery, avoiding tissue damage and the risk of additional morbidity [7]. The main disadvantage of these membranes is the unpredictable reabsorption time and the degree of degradation [13]. It has been described that increased degradation of collagen membranes is mainly caused by the enzymatic activity of infiltrating macrophages and polymorphonuclear leukocytes [11]. Among the different resorbable membranes available, collagen membranes are the most widely studied and investigated [6].

Although the clinical application of collagen membranes in bone regeneration is well established, only a limited number of studies have investigated their resorption patterns. These studies have stated that the degradation of collagen membranes can be produced between 4 days and 6 weeks after surgical placement [14]. Collagen membranes have been shown to exhibit excellent biocompatibility, but rapid resorption. When tested in a rabbit calvarium model, different degradation patterns have been described depending on the origin of the collagen membranes [15]. They may come from the tendon, skin, pericardium, or other regions of purified bovine, equine or porcine collagen [16]. Membranes made from porcine collagen have an open and porous collagen network, but also dense. The inherent open pores of native porcine skin facilitate the migration of blood vessels into the defect area, allowing for rapid vascularization of the underlying wound bed, while the density of the membrane maintains a barrier against ingrowth of soft tissue [16]. The collagen fibers in tendons are predominantly oriented longitudinally and, to a lesser extent, also transversely and horizontally. Furthermore, tendon collagen fibers are crimped, in contrast to skin fibers, which are even fibers [17]. The pericardium has to withstand the forces of the heart muscle; it has an exceptionally dense collagen-like structure, which gives it rigidity and multidirectional tear resistance [17]. In addition, equine tendon collagen retains a partial lateral packing, thanks to the higher content of lysine and hydroxylysine compared to other mammal tendon collagens. This feature explains why collagen and collagen-based devices from horse tendons may be intrinsically more resistant to degradation [18].

Differences in collagen tissue structure lead to different degradation responses and clinical outcomes [15,19]. It has also been suggested that other characteristics may influence the degradation behavior of a membrane, such as porosity, thickness and weight. The thickness and weight will also affect the mechanical properties of the membrane. Porosity allows nutrients infiltration into the defect, which promotes bone growth, although excessively large pores can make the membranes less effective as a barrier against soft tissue cells [11,20]. Variations in composition, structure and properties will definitely influence the degree of degradation. The reabsorption rate of commercially available collagen membranes can vary broadly. For this reason, it is important to choose a membrane that maintains its structural integrity and mechanical properties for long enough to permit the proliferation and maturation of the desired cells within the wound [5]. Performing in vitro studies will allow for detecting the influence of the composition of the membrane and their properties in the duration of the degradation process. Hence, in the present study, the aim was to investigate the biodegradation pattern of five collagen membranes from different origin, in a period of 1 to 50 d. A qualitative microstructural evaluation and a quantitative analysis of collagen membrane degradation were performed. The null hypotheses that were tested were that: (i) the five membranes for guided bone regeneration do not present the same degradation pattern over time; and (ii) the five membranes do not resist the different degradation processes in a similar way (hydrolytic, bacterial collagenase and enzyme resistance).

## 2. Materials and Methods

### 2.1. Membranes Description

Five experimental guided bone regeneration (GBR) collagen membranes were tested. Membranes are commercially available and CE-certified for oral applications and all have heterologous origin. The 0.2 mm-thickness resorbable membranes investigated were: (1) Biocollagen (Bioteck by Bioteck S.p.A, Torino, Italy); (2) Heart (Bioteck by Bioteck S.p.A, Torino, Italy); (3) Evolution X-fine (Osteobiol by Tecnoss, Torino, Italy); (4) CopiOs (Zimmer Biomet Dental, FL, USA); and (5) Parasorb Resodont (Resorba by Resorba Medical GmbH, Nürnberg, Germany). According to the manufacturers, all of the resorbable membranes used in the present study must be previously hydrated. Biocollagen is derived from equine type I collagen obtained from the Achilles tendon. Heart is an equine pericardium membrane, where molecular links among its constituents (collagen and elastin) are preserved. Evolution X-fine is a resorbable pericardium porcine membrane obtained from heterologous mesenchymal tissue; its structure is made of dense collagen fibers. CopiOs is a membrane made from the bovine pericardium based on type I and III collagen. Finally, Parasorb Resodont is a resorbable membrane made of natural cross-linked equine collagen fibers without chemical additives. The main characteristics of the membranes used in this biodegradation study are listed in Table 1.

### 2.2. Degradation Assays

Membranes samples were cut to a size of 10 × 10 mm^2^. Three specimens of each membrane type were employed for each degradation test (*n* = 3). Three different degradation tests were performed [1,11].

(1) Hydrolytic degradation test: membranes were immersed in phosphate buffer saline solution (PBS) at 37 °C [21].

(2) Enzyme resistance test: membranes were submerged in a 0.25% porcine trypsin solution (Sigma-Aldrich, St. Louis, MO, USA) and were incubated at 37 °C [6].

(3) Bacterial collagenase resistance test: a collagenase solution from *Clostridium histolyticum* bacteria Type V (Sigma-Aldrich, St Louis, MO, USA) was used to submerge the membranes. It is a mixture of several different enzymes containing; collagenase, non-specific proteases, clostripain, neutral protease, and aminopeptidase activities. These enzymes act together to cause a breakdown of tissues. The specific activity is ≥125 CDU/mg solid. A collagenase concentration of 2 IU/mL in 50 mM Tris HCl (pH 7.4), containing 10 mM CaCl_2_, was used [22,23].

After each 48 h, degradation solutions were removed carefully through suction and renewed [24].

Membranes were retrieved from the media after the following time periods: 1 h, 6 h, 24 h, 48 h, 7 d, 14 d, 28 d and 50 d. After each period of immersion, membranes were completely dried in a vacuum chamber. Weight (W) was then measured with an analytic balance (A&D-Instruments, Frankfurt, Germany) with an accuracy of 0.0001 g; the complete device was mounted on an antivibratory table. Thickness (Th) was also measured at random positions by means of a digital caliper (Mitutoyo 293-561, Tokyo, Japan) [1,11].

### 2.3. Statistical Analysis

The normal distribution of data was established by the Kolmogórov–Smirnov test (*p* > 0.05). Therefore, parametric tests were employed. Multiple ANOVA models were used to assess the influence of the independent variables (degradation solution, type of matrix and immersion time) on the dependent variables (weight and thickness). Analyses of interactions were also carried out. ANOVA and Student–Newman–Keuls post hoc comparisons were performed to determine differences between materials and degradation solutions. To permit these comparisons, the variables weight and thickness were converted into the percentage of variation with respect to the initial measurement following the equation:

Percentage of loss = [(*X*_0_ − *X*_t_)/*X*_0_] × 100, (1)

where *X*_0_ is the initial weight or thickness of the specimen, and *X*_t_ is the specimen’s weight or thickness at each time-point (t).

Pairwise comparisons were performed to ascertain for differences between immersion time-points within the same membrane and solution experimental group. Significance was always considered at *p* < 0.05, except for pairwise comparisons, where a Bonferroni’s correction was applied and *p* < 0.001 was set. Statistical analysis was performed using SPSS 25.0 (SPSS Inc., Chicago, IL, USA) software package.

### 2.4. Light Microscopy Analysis

At the end of the storage period of 7 d, specimens were observed under a stereomicroscope Olympus SZ-60 (Olympus, Tokyo, Japan) for microstructural analysis. Images were taken at 60× and 120× magnifications.

## 3. Results

**Figure 1:**
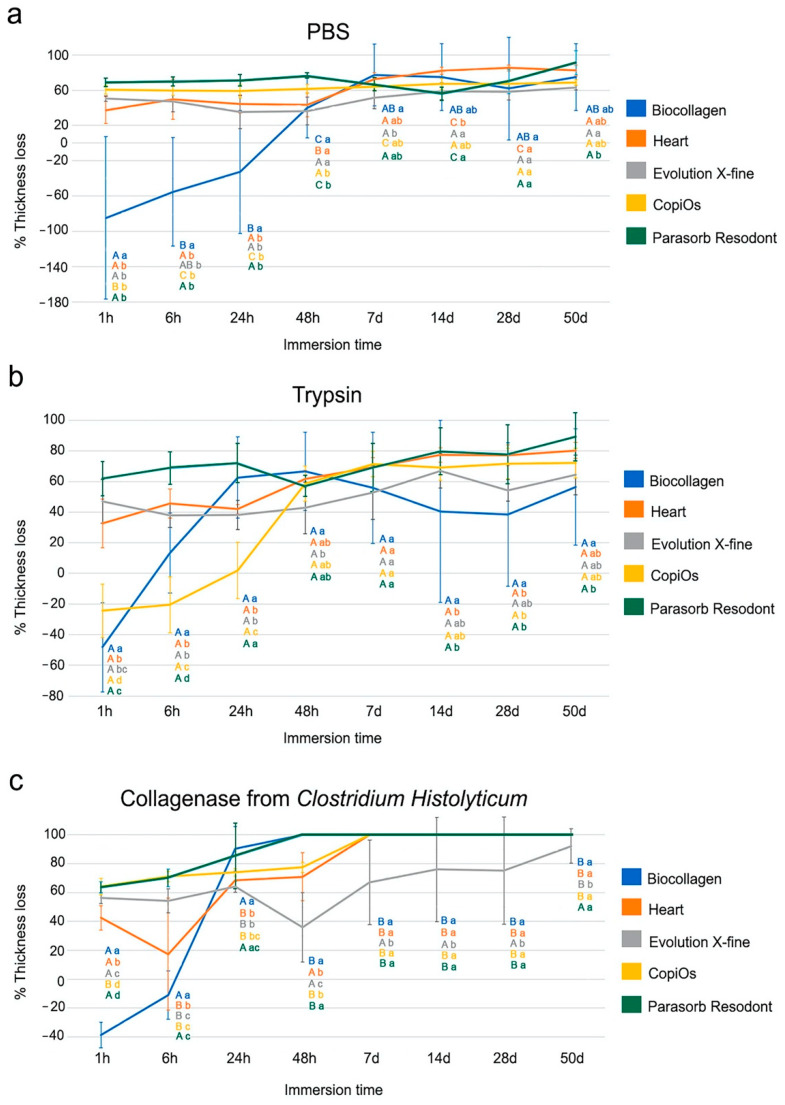
Percentage of thickness loss of the five tested membranes after being submitted to (**a**) PBS, (**b**) trypsin and (**c**) *C. histolyticum* collagenase over the different time-points. Values are expressed as mean and standard deviation. Significant differences among membranes are shown with low-case letters and capital letters indicate differences between the different degradation tests within the same membrane. Student-Newman-Keuls tests were performed for multiple comparisons (*p* < 0.05).

The thickness loss values of the membranes, expressed in percentage, across the different degradation tests and times, are represented in Figure 1. The thickness values in mm of the five tested membranes (Biocollagen, Heart, Evolution X-fine, CopiOs and Parasorb Resodont) into the different solutions (PBS, trypsin and *C. histolyticum* collagenase) over the different immersion time periods (1 h, 6 h, 24 h, 48 h, 7 d, 14 d, 28 d and 50 d) are shown in Table 2. Comparisons between membranes within the same solution are expressed in Figure 1, while the loss of thickness after each immersion time-point compared to the initial value of each of the membranes type is shown in Table 2.

### 3.1. Thickness Evaluation after PBS Degradation Assay

The comparison of the thickness loss, expressed as a percentage, suffered by membranes submitted to the PBS degradation assay is shown in Figure 1a. At 1 h, Biocollagen’s thickness increased by more than 80% due to water absorption during first immersion, while the rest of membranes experienced a marked loss of thickness of over 30% (Figure 1a). After 24 h of storage, all membranes suffered a reduction in Th percentage, with Biocollagen being the membrane whose weight loss values were lower. The trend was as follows: Parasorb Resodont > CopiOs > Heart > Evolution X-fine > Biocollagen. For Parasorb Resodont, CopiOs, Heart and Evolution X-fine, the trend was maintained, and the thickness loss values were similar at 24 and 48 h. However, Biocollagen from 24 to 48 h suffered a marked reduction in thickness, with a loss of over 70%. At the 7 d time-point, Biocollagen attained the highest and Evolution X-fine the lowest percentage thickness values; the trend was as follows: Biocollagen > Heart > Parasorb Resodont > CopiOs > Evolution X-fine (Figure 1a). All membranes ended 50 d of storage with more than 50% thickness loss. The final trend was: Parasorb Resodont > Heart > Biocollagen > CopiOs > Evolution X-fine (Figure 1a).

In general terms, the five membranes had an increasing loss of thickness over different time-points when comparing to the initial, except for Biocollagen, which at 1 h did not experience any loss of thickness (Table 2). None of the membranes were completely degraded in this medium. However, all membranes ended 50 d of storage with lower thickness values than the initial value (Table 2).

### 3.2. Thickness Evaluation after Trypsin Degradation Assay

The percentage of thickness loss suffered by the different membranes studied when they were immersed in trypsin is shown in Figure 1b. At the 1 h time-point, Biocollagen and CopiOs did not suffer loss of percentage thickness values, experiencing a gain of over 40% and 20%, respectively. At the same time-point, Heart, Evolution X-fine and Parasorb Resodont showed a loss of Th values of over 30% (Figure 1b). From 1 to 48 h, Biocollagen and CopiOs suffered a marked loss of thickness. The rest of the membranes suffered a gradual loss of Th, with values very similar to those obtained in the first moment of immersion. After 7 d of storage, Parasorb Resodont, Heart and CopiOs performed similarly, with the highest loss of percentage thickness values, while Biocollagen reached intermediate performance and Evolution X-fine attained the lowest loss of percentage thickness values (Figure 1b). At 14 and 28 d, the trend was as follows: Parasorb Resodont ≥ Heart > CopiOs > Evolution X-fine > Biocollagen. After 50 d of immersion, all membranes suffered a reduction in percentage thickness of above 50%. The trend was as follows: Parasorb Resodont > Heart > CopiOs > Evolution X-fine > Parasorb Resodont (Figure 1b).

In general terms, all membranes suffered changes in their thickness throughout the different time-points (Table 2). All membranes endured 50 d of storage, suffering a statistically significant loss of thickness (Figure 1b).

### 3.3. Thickness Evaluation after C. histolyticum Collagenase Degradation Assay

The comparison of the thickness loss, expressed as a percentage, suffered by membranes submitted to collagenase degradation assay is shown in Figure 1c. At the 1 h time-point, CopiOs attained the highest loss of percentage thickness values, close to 70%, this being similar to the loss experienced by Parasorb Resodont. Heart and Evolution X-fine experienced a thickness loss between 40 and 60%. Finally, Biocollagen did not experience a loss of thickness; instead, its thickness increased by almost 40%. The trend was as follows: CopiOs ≥ Parasorb Resodont > Evolution X-fine > Heart > Biocollagen (Figure 1c). After 48 h of storage, Parasorb Resodont and Biocollagen completely degraded. After 7 d of immersion, Heart and CopiOs totally degraded, with a 100% reduction in Th values (Figure 1c). Evolution X-fine was the only membrane which overcame 50 d of storage with high loss of percentage thickness values of nearly 90%.

In general terms, this degradation assay was the most dangerous to the membranes studied (Table 2). All membranes suffered a marked reduction in their thickness values over time when compared to the initial time-point (Table 2). Parasorb Resodont, Biocollagen, Heart and CopiOs could not endure more than 7 d of storage, while Evolution X-fine reached 50 d of immersion with low thickness values.

**Figure 2:**
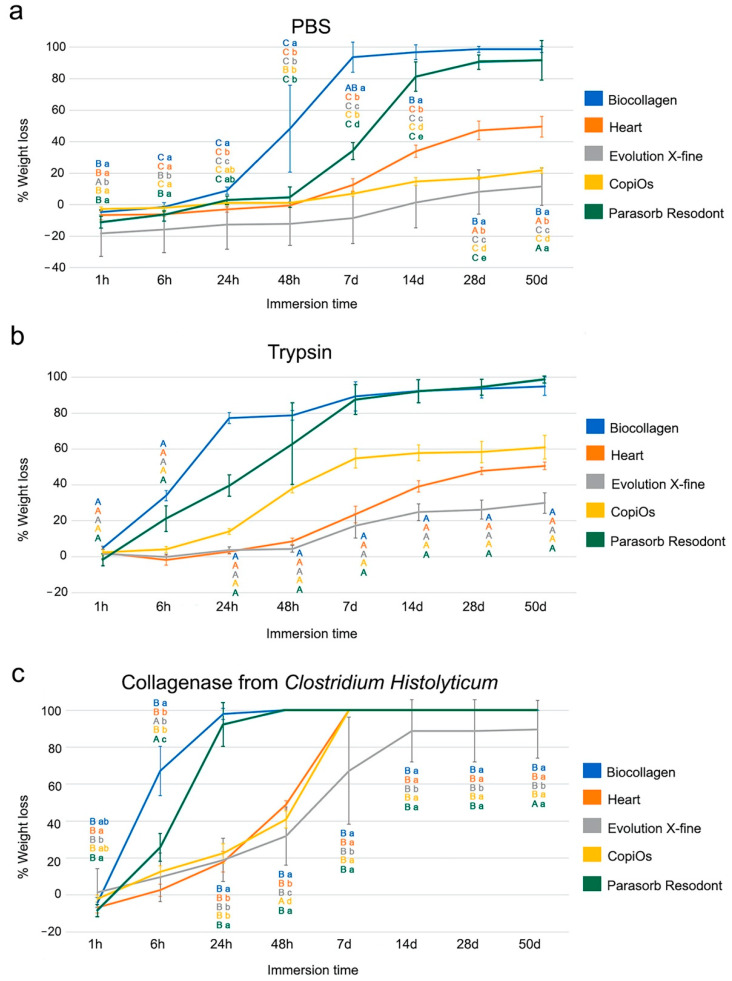
Percentage of weight loss of the five membranes studied submitted to: (**a**) PBS, (**b**) trypsin and (**c**) C. histolyticum collagenase over different time-points. Values expressed as mean and standard deviation. Significant differences among membranes are shown with lowercase letters and with capital letters for differences between degradation tests within the same membrane. Student-Newman-Keuls tests were performed for multiple comparisons (*p* < 0.05).

The weight (W) loss values, expressed in percentage, over distinct degradation tests and times, are shown in Figure 2. The weight (W) values in grams of the five membranes studied (Biocollagen, Heart, Evolution X-fine, CopiOs and Parasorb Resodont) subjected to different solutions (PBS, trypsin and *C. histolyticum* collagenase) during various submersion time periods are presented in Table 3. Comparisons between membranes within the same solution are shown in Figure 2, while the weight after each immersion time-point and comparisons with initial weight values are displayed in Table 3.

### 3.4. Weight Evaluation after PBS Degradation Assay

The weight loss percentage suffered by membranes submitted to PBS degradation assay is shown in Figure 2a. From the first immersion period up to the 6 h time-point, membranes hardly suffered from weight loss. The highest loss of weight corresponded to CopiOs at the 1 and 6 h time-points, this being similar to Biocollagen after 6 h of storage. From 24 to 48 h, Biocollagen suffered a marked loss of weight, while the rest of the membranes maintained their weight values. At the 48 h time-point, Evolution X-fine attained the lowest and Biocollagen the highest loss of percentage weight values. The trend was as follows: Biocollagen > Parasorb Resodont > CopiOs > Heart > Evolution X-fine (Figure 2a). After 7 d of immersion, this trend changed as follows: Biocollagen > Parasorb Resodont > Heart > CopiOs > Evolution X-fine, and it was maintained until 50 d of storage (Figure 2a). So, at the end of the study, Biocollagen and Parasorb Resodont experienced a loss of weight of above 90%, Heart above 40%, CopiOs above 20% and Evolution X-fine experienced an almost 10% weight loss.

In general terms, all membranes suffered significant weight loss after completing the different study periods, except for Evolution X-fine, which never attained significance (Table 3). After 50 d of storage, Biocollagen reached the highest loss of percentage weight values, and Evolution X-fine the lowest (Figure 2a).

### 3.5. Weight Evaluation after Trypsin Degradation Assay

The comparison of the weight loss, expressed as a percentage, suffered by membranes submitted to collagenase degradation assay is shown in Figure 2b. After 1 h of immersion, Biocollagen attained the highest loss of percentage weight values, but these values were below 10%. At this time-point, the trend was as follows: Biocollagen > CopiOs > Evolution X-fine ≥ Heart > Parasorb Resodont (Figure 2b). At the 48 h and 7 d time-points, the highest loss of weight corresponded to Biocollagen, followed by Parasorb Resodont. CopiOs and Heart performed intermediately, and Evolution X-fine showed the lowest loss of percentage weight values. After 28 d of storage, the trend was as follows: Parasorb Resodont ≥ Biocollagen > CopiOs > Heart > Evolution X-fine. This trend was maintained until the final stage of study (Figure 2a). At 50 d of immersion, Parasorb Resodont and Biocollagen attained the highest loss of weight values, these being over 90%. CopiOs and Heart performed intermediately, with nearly 60% and 50% weight loss. Finally, Evolution X-fine exceeded 50 d of storage, with weight loss close to 30%.

In general terms, all membranes showed significant weight loss over time in the trypsin degradation assay after completing the different study periods (Table 3).

### 3.6. Weight Evaluation after C. histolyticum Collagenase Degradation Assay

The percentage of weight loss suffered by membranes when immersed in *C. histolyticum* is shown in Figure 1c. At the 6 h time-point, all membranes experienced a loss of weight, this value being higher for Biocollagen, followed by Parasorb Resodont, CopiOs, Evolution X-fine and, finally, Heart. After 24 h of storage, the highest loss of percentage weight values corresponded to Biocollagen and the lowest to Heart. The trend found at this time-point was as follows: Biocollagen > Parasorb Resodont > CopiOs > Evolution X-fine > Heart (Figure 2c). At the 48 h time-point, Biocollagen and Parasorb Resodont were completely degraded. After 7 d of storage, CopiOs and Heart were fully degraded. Evolution X-fine, with a loss of percentage weight of close to 90%, overcame 50 d of immersion (Figure 2c).

In general terms, the bacterial collagenase solution was found to be the most aggressive test, causing the complete degradation of all membranes before 7 d. All membranes experienced changes in their weight over time, when compared to the initial values (Table 3).

### 3.7. Matrices Morphological Analysis

**Figure 3:**
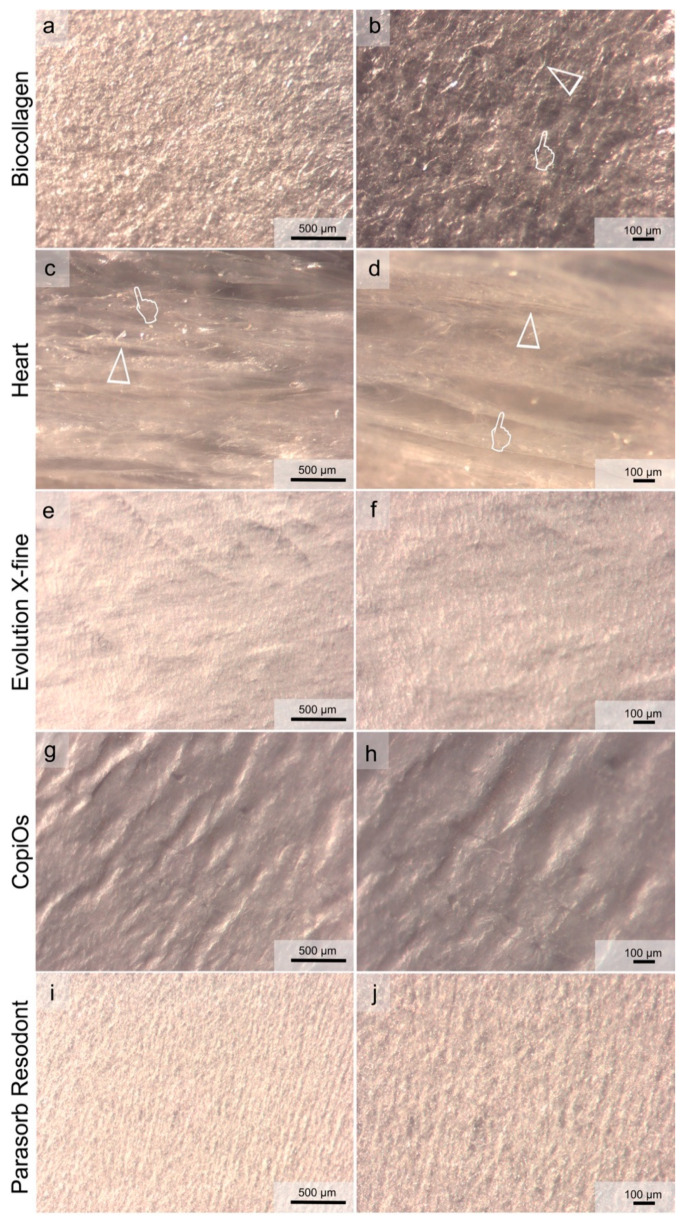
Light micrographs taken of the smooth surface of the membranes before immersion. (**a**) Biocollagen surface at 60× magnification. (**b**) Biocollagen at 120× magnification. (**c**) Heart surface at 60× magnification. (**d**) Heart at 120× magnification. (**e**) Evolution X-fine surface at 60× magnification. (**f**) Evolution X-fine surface at 120× magnification. (**g**) CopiOs surface at 60× magnification. (**h**) CopiOs surface at 120× magnification. (**i**) Parasorb Resodont surface at 60× magnification. (**j**) Parasorb Resodont surface at 120× magnification. In (**a**,**c**,**e**,**g**,**i**), the scale bar is 500 µm, and it is 100 µm in (**b**,**d**,**f**,**h**,**j**). Pointers indicate pores of different size across the membrane’s surface. Arrow heads indicate collagen fibers.

**Figure 4:**
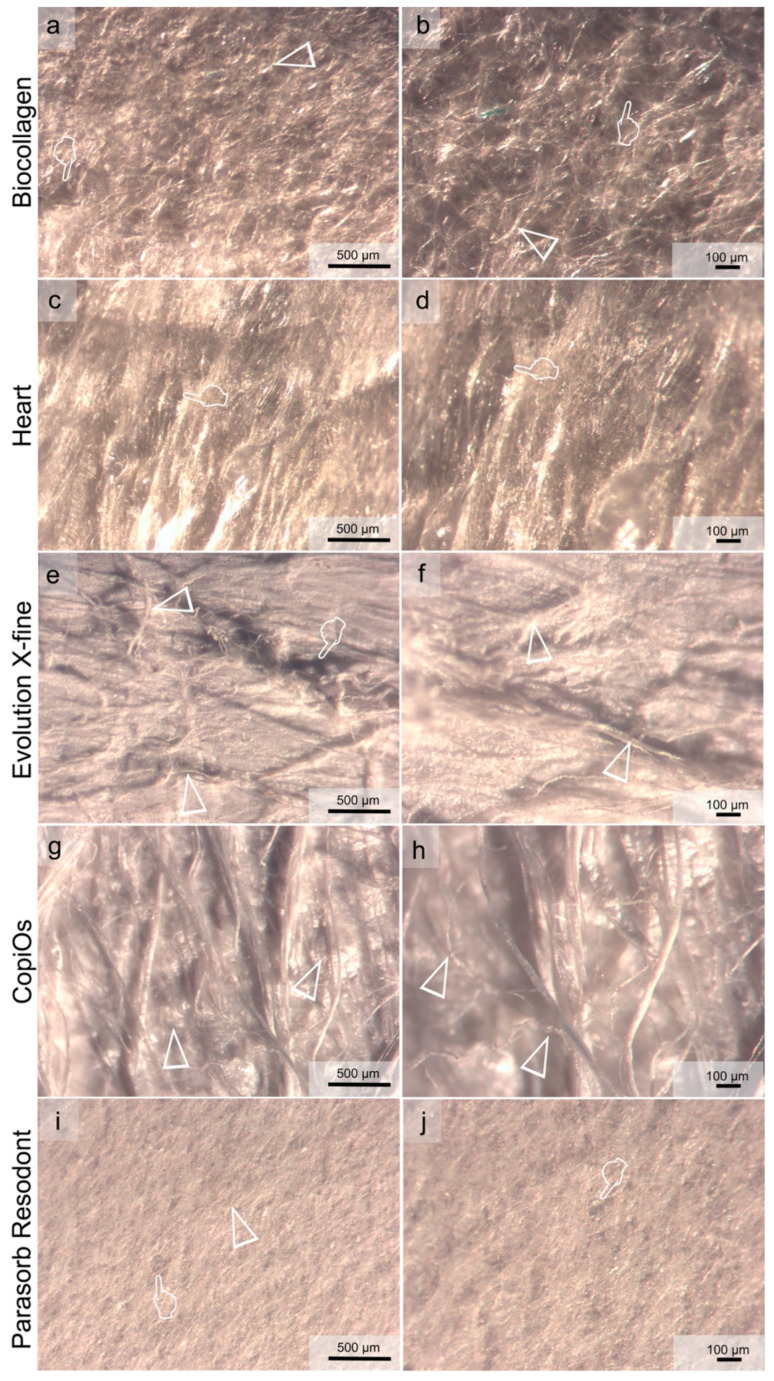
Light micrographs taken of the rough surface of the membranes before immersion. (**a**) Biocollagen surface at 60× magnification. (**b**) Biocollagen at 120× magnification. (**c**) Heart surface at 60× magnification. (**d**) Heart surface at 120× magnification. (**e**) Evolution X-fine surface at 60× magnification. (**f**) Evolution X-fine surface at 120× magnification. (**g**) CopiOs surface at 60× magnification. (**h**) CopiOs surface at 120× magnification. (**i**) Parasorb Resodont surface at 60× magnification. (**j**) Parasorb Resodont surface at 120× magnification. In (**a**,**c**,**e**,**g**,**i**), the scale bar is 500 µm, it is and 100 µm in (**b**,**d**,**f**,**h**,**j**). Pointers indicate pores of different size across the membrane’s surface. Arrow heads indicate collagen fibers. Arrows point to mineral deposits on the membrane’s surface.

Figure 3 and Figure 4 show light micrographs taken from the different surfaces of membranes tested before being submitted to immersion in the degradation solutions. GBR collagen membranes have two different surfaces: one smooth and the other rough. Compact collagen fibers with cellular occlusive properties constituted a smooth surface (Figure 3). Evolution X-fine, CopiOs and Parasorb Resodont presented a profile of dense distribution of filaments with fewer detectable pores (Figure 3e–j). Surfaces with randomly distributed collagen fibers in Biocollagen (Figure 3a,b) and with a trabecular structure in Heart (Figure 3c,d) were observed, respectively. The second layer, presented in Figure 4, consisted of a rough collagen structure designed to be positioned in direct contact with the bone surface or the bone defect. Parasorb Resodont showed a semi-compact collagen surface where the pores and the collagen fibers were hardly visible (Figure 4i,j). Biocollagen had collagen fibers that were absorbed throughout the surface of the membrane formed by lattices and pores aiming to allow cell adhesion (Figure 4a,b). Heart, Evolution X-fine and CopiOs presented collagen fibers arranged in layers that randomly run parallel to the surface. Light microscopy clearly showed regions with larger pores (more than 100 μm) located in areas where collagen fibrils were absent.

**Figure 5:**
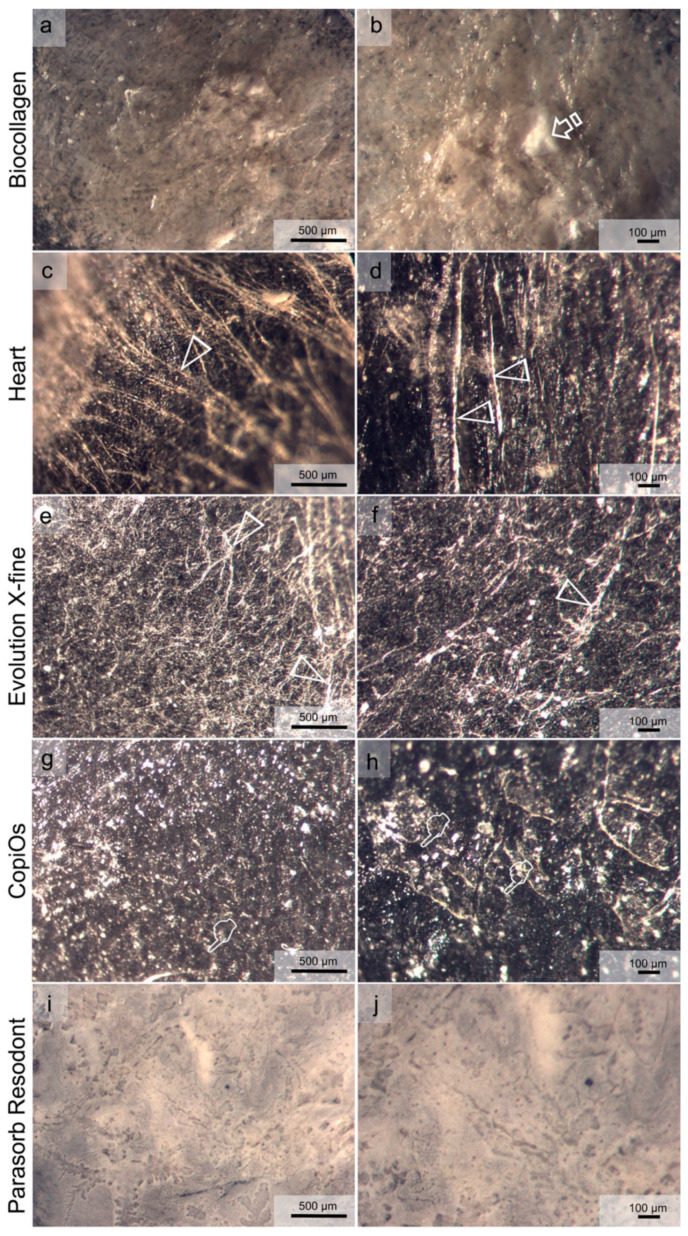
Light micrographs taken of membranes after immersion in the PBS degradation test. (**a**) Biocollagen surface at 60× magnification. (**b**) Biocollagen at 120× magnification. (**c**) Heart surface at 60× magnification. (**d**) Heart surface at 120× magnification. (**e**) Evolution X-fine surface at 60× magnification. (**f**) Evolution X-fine surface at 120× magnification. (**g**) CopiOs surface at 60× magnification. (**h**) CopiOs surface at 120× magnification. (**i**) Parasorb Resodont surface at 60× magnification. (**j**) Parasorb Resodont surface at 120× magnification. The scale bar is 500 µm in (**a**,**c**,**e**,**g**,**i**), and 100 µm in (**b**,**d**,**f**,**h**,**j**). Pointers indicate pores of different size across the membrane’s surface. Arrow heads indicate collagen fibers. Arrows point to mineral deposits on the membrane’s surface.

**Figure 6:**
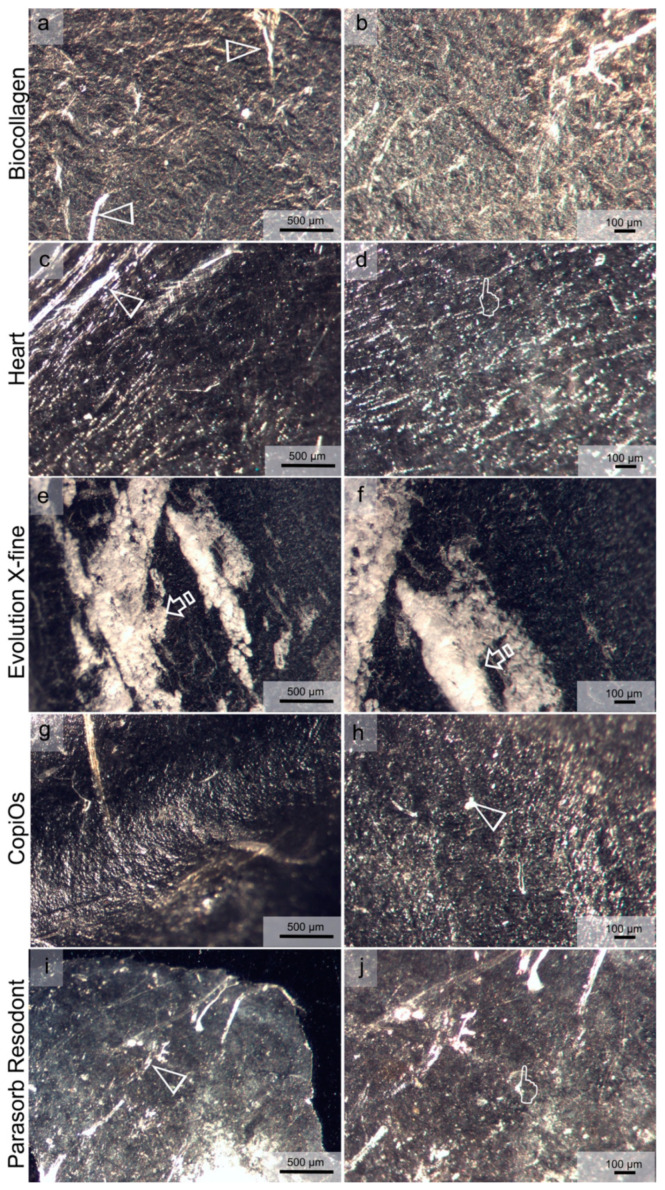
Light micrographs taken of membranes after immersion in the Trypsin degradation test. (**a**) Biocollagen surface at 60× magnification. (**b**) Biocollagen at 120× magnification. (**c**) Heart surface at 60× magnification. (**d**) Heart surface at 120× magnification. (**e**) Evolution X-fine surface at 60× magnification. (**f**) Evolution X-fine surface at 120× magnification. (**g**) CopiOs surface at 60× magnification. (**h**) CopiOs surface at 120× magnification. (**i**) Parasorb Resodont surface at 60× magnification. (**j**) Parasorb Resodont surface at 120× magnification. The scale bar is 500 µm in (**a**,**c**,**e**,**g**,**i**), and 100 µm in (**b**,**d**,**f**,**h**,**j**). Pointers indicate pores of different sizes across the membrane’s surface. Arrow heads indicate collagen fibers.

Figure 5 and Figure 6 display the appearance of the membranes after 7 days of immersion in PBS and Trypsin. The effect of the membrane degradation prevented us from distinguishing rough from smooth membrane surfaces in both degradation tests. After immersion in PBS, Biocollagen showed a dense surface without appreciation of collagen fiber distribution, but some mineral deposits were deposited on its surface (Figure 5a,b). Multiple bundles of collagen of different sizes were aligned in parallel and were separated from each other by irregular spaces in Heart, Evolution X-fine and CopiOs. Parasorb Resodont showed a regular surface where pores and collagen fibers were hardly visible (Figure 5i,j). After the trypsin test, Biocollagen demonstrated less uniformity regarding collagen fiber orientation, with a random distribution of collagen fibers (Figure 6a,b). Heart presented multiple bundles of collagen distributed in layers (Figure 6c,d). The fiber network was difficult to appreciate in the surface of Evolution X-fine, CopiOs and Parasorb Resodont. A large mineral condensation was found on Evolution X-fine’s surface (Figure 6e,f).

## 4. Discussion

The present study examined the effect of different immersion media on the biodegradation pattern of five commercially available membranes over time. The presented data demonstrated that the membranes suffered a continuous degradation process throughout the multiple storage periods and did not resist to degradation assays equally. Evolution X-fine was the most resistant membrane to the degradation testing, assessed by weight and thickness (Figure 1 and Figure 2).

Biocollagen, Heart, Evolution X-fine, CopiOs and Parasorb Resodont are resorbable membranes mainly composed of collagen. The donor area and the origin of collagen vary among membranes; this may explain the differences between degradation behavior [15]. Measured by weight, Biocollagen obtained the highest percentage of degradation in the initial periods (Figure 2). However, observing the results presented for thickness, Biocollagen did not suffer a loss of thickness until 6 or 24 h of storage. Furthermore, the final thickness loss of Biocollagen was less than in the other membranes (Figure 1). Parasorb Resodont degraded by almost 90%, reaching the highest percentages of thickness loss after 50 d of immersion in PBS and trypsin, and it was completely degraded after 7 days in *C. histolyticum* collagenase solution. Signs of degradation showed that the loss of the membranes’ structural integrity occurred before even 6 h. Our results agree with previous findings of Toledano et al. [11], who demonstrated that collagen membranes submitted to degradation tests attained a significant reduction in thickness and weight before 4 h of immersion. Zhao et al. [25] reported a disintegration of membrane material following 3 weeks of implantation in rats’ subcutaneous pouches. In addition, Calciolari et al. [14] clinically applied collagen membranes for GBR and found a significant reduction in membrane thickness from 7 to 30 days of healing. One possible reason for this performance is that both Biocollagen and Parasorb Resodont are made of equine collagen. Gallo et al. [18] stated that collagen from horses is the most resistant to degradation. In contrast, in the present research, it has been observed that among all of the membranes evaluated, those of equine origin demonstrated faster degradation. In addition, Heart, an equine collagen membrane, performed intermediate in loss of weight and thickness values, being the third membrane with the highest degradation percentages. In accordance with our findings, Toledano et al. [11] submitted three membranes, two from equine origin and one from porcine origin to different degradation media. Derma Fine, a porcine collagen-based membrane, demonstrated the greatest resistance to all degradation challenges under in vitro conditions [11]. Differences in membranes degradation may be attributed to variations in membrane composition [15,19]. Thus, the first null hypothesis has to be rejected.

Guided bone regeneration (GBR) is based on the concept of cell exclusion and space maintenance to allow sufficient time for new bone formation [1]. For regeneration to occur, the membrane is expected to remain intact up to 4–6 months [18]. Evolution X-fine performed as the most resistant membrane to the degradation tests followed by CopiOs. In addition to their origin, porcine and bovine, respectively, these membranes presented the least porous surface when they were evaluated with optical microscopy before immersion (Figure 3 and Figure 4). Morphological changes have been observed on collagen matrixes after degradation. These are the disarrangement of the collagen net and pore enlargement through the widening of the interfibrillar spaces. These findings are consistent with previously described alterations that were found after water degradation of human collagen [26]. Previous studies correlating the membrane structure with degradation reported that thicker collagen fibers, higher collagen density and a more compact structure may be produce slower biodegradation [27,28].

When comparing membranes surface, Evolution X-fine surface was smoother and more homogenous to any other membrane type regardless of the side observed. A less porous membrane characteristic seems to avoid excessive humidity and the consequent loss of the physical properties of the membrane [29]. It is generally accepted that the absorption ability of barrier membranes greatly varies depending on their origin and composition [30]. Therefore, the macroscopic structure and origin of these membranes explains their greater resistance to degradation. Caballé-Serrano et al. [19] found that porcine origin barrier membranes and porous membranes showed a high wettability. In addition, Toledano et al. [11] also observed that a specific distribution of the collagen fibers and the presence of pores influenced the degradation behavior. Evolution X-fine, made from collagen fibers from porcine pericardium, attained a reduction of less than 50% in thickness and 20% in weight, for the media PBS and trypsin (Figure 1 and Figure 2). Measuring the weight and thickness of the membrane after different immersion times makes it possible to quantitatively evaluate how a membrane behaves in a medium. Thickness features could lead to a remarkable interpretation, since the barrier effect depends on the ability to maintain a minimum thickness for the full period, enabling the desired cell exclusion. Several studies have investigated whether the addition of a second membrane layer prevents rapid reabsorption and maintains the essential function of the membrane [1,15]. The obtained results show that collagen degrades at the same rate; however, having a double thickness results in a significantly greater amount of residual collagen [1,15]. This makes possible to extend the functional time of GBR membranes. Consequently, in addition to the origin and macroscopic structure, the initial thickness of the membrane is also an aspect to be considered.

The main disadvantages of collagen-based membranes are their lack of rigidity and fast degradation kinetics by endogenous collagenases, so that the barrier function may not remain long enough for tissue regeneration [31]. From 0 to 1 h of immersion, weight and thickness values increased for Biocollagen. This could be explained by the major water absorption that likely occurs during the first immersion. Biocollagen comes from equine tendons, where collagen fibrils are coupled via proteoglycans (PGs) linkages [32]. PGs are one type of non-collagenous protein, and primarily contain a core protein and glycosaminoglycans (GAGs). GAGs are highly polar and negatively charged, thus having a strong tendency towards attracting water molecules into the matrix. On the other hand, water also functions as a plasticizer, thus diminishing mechanical properties and facilitating the degradation process [33].

In the present study, *C. histolyticum* was the most aggressive medium for membrane degradation, as four out of five collagen membranes could not endure more than 7 d of storage (Figure 1c and Figure 2c). Evolution X-fine was the only membrane that reached 50 d of immersion, but with a great rate of resorption in *C. histolyticum* (Table 2 and Table 3). The rapid degradation of collagen membranes in this medium has been previously verified by other authors, Vallecillo et al. [34] observed early degradation when three collagen matrices were submitted to the *C. histolyticum* assay. Fibro-Gide, a cross-linked collagen matrix, was uniquely able to withstand 7 d of storage in a bacterial collagenase test. The authors attributed the greater resistance of this matrix compared to the others studied to the cross-linking of collagen. Cross-linked methods have been proposed in order to decelerate collagen-based membrane resorption [10,25,26]. Toledano et al. [11] showed that samples subjected to *C. histolyticum* challenge lost more thickness and weight than those in trypsin and PBS. Degradation in bacterial collagenase in vitro is an adequate test to assess and predict membrane stability in the presence of wound dehiscence [18]. Sela et al. [17] faced collagen membranes for GBR to different enzymes produced by periodontopathic bacteria. They suggested that bacterial collagenase is more aggressive in degrading collagen membranes than the proposed enzyme cocktail [17]. Compared to the enzyme and hydrolytic degradation assays, bacterial collagenase has been shown to be more effective in degrading collagen; all membranes suffered an increased loss of thickness and weight, regardless of their origin [11]. When the samples were immersed in PBS and Trypsin, loss was experienced, but they all endured 50 d of immersion. However, in *C. histolyticum*, membranes were not able to resist degradation beyond 7 d, except for Evolution X-fine. For this reason, the second null hypothesis has also rejected.

The present study demonstrates the in vitro degradability behavior and morphological analysis of five commercially available collagen-based membranes from different heterologous origins. This in vitro study allows for detecting the influence of the initial composition and origin of the collagen membrane in the biodegradation pattern, regardless of the cellular reaction of the recipient organism. The present results may be useful to surgeons to better select a GBR membrane according to the procedure to be performed and the risk of wound dehiscence. Evolution X-fine is made from porcine pericardium collagen and has shown the highest resistance to the tested degradation challenges, even in collagenase from *C. histolyticum*. This superior resistance added to a more homogeneous surface prolongs the barrier effect of the membrane, even in case of being exposed to a more aggressive environment. Therefore, Evolution X-fine should be considered as a potential candidate for use in those GBR procedures prone to cross-infection and in several challenging pathologies in which bacteria may alter the positive clinical outcome, including periodontal diseases [35].

Three degradation tests were performed, which represent more aggressive conditions for collagen membranes than just submerged healing. This allows the membranes to be evaluated in the worst scenario, which may occur clinically in the presence of wound dehiscence. In clinical practice, it is common to observe membrane exposure, especially during the early stages of healing [14]. As the membrane is exposed to the oral environment, saliva and bacteria will trigger more rapid degradation of the biomaterial, compromising the success of bone regeneration [14].

Nevertheless, the results of this study should be considered with caution due to its limitations. The correlation between membranes’ topography, collagen fibrils’ nanostructure and mechanical properties was not studied. Further research will be conducted in order to ascertain whether these changes in collagen nanostructure may also be related to a decrease in the mechanical properties of collagen membranes through the ongoing degradation process. Additionally, the impact of embedded cells on membrane degradation is missing. Despite the limitations of the study, our in vitro findings are intended to give a first estimation of the membranes behavior, but it should also be considered that in vivo, a more complex set of different cell types, body fluids and possible microbial contamination may influence the membrane degradation process, with the oral cavity environment being an scenario that is difficult to mimic [27,28]. Therefore, it might be challenging to directly transfer these data to the clinical practice. Authors encourage researchers to continue evaluating how to retard membranes resorption. Membranes doped with antibacterial agents could also be prepared and studied aiming to counteract the rapid degradation in the presence of bacterial enzymes.

## 5. Conclusions

Evolution X-fine collagen membrane from porcine pericardium origin has been proven to be the least affected when immersed in PBS, trypsin and *C. histolyticum*. Biocollagen and Parasorb Resodont, both of equine origin, experienced the greatest degradation, measured in thickness and weight, under in vitro conditions. The bacterial collagenase solution acted as the most aggressive test against membranes, producing complete degradation of four membranes before 7 days of immersion. The loss of structural integrity and pores is presented after storage processes, demonstrating the occlusive effect of GBR membranes to exclude the epithelium and connective tissue.

## Figures and Tables

**Table 1:** Commercial name, collagen type, collagen origin and clinical characteristics (cross-link, estimated duration of the barrier effect) of the tested collagen membranes. The information was obtained from the membrane providers.

Membrane Comercial Name	Collagen Type	Origin	Cross-Link	Estimated Barrier Effect Duration
*Biocollagen*	Collagen type I	Equine tendon	No	4–6 weeks
*Heart*	NR	Equine pericardium	No	12–16 weeks
*Evolution X-fine*	Collagen fibers	Porcine pericardium	No	8 weeks
*CopiOs*	Collagen type I	Bovine pericardium	No	16–24 weeks
*Parasorb Resodont*	Collagen fibers	Equine	Natural	NR

NR: not reported.

**Table 2:** (**a**) Thickness values (mm) of the five membranes (Biocollagen, Heart, Evolution X-fine, CopiOs and Parasorb Resodont) after being submitted to: PBS, trypsin and *C. histolyticum*, from 1 h to 50 d. Values are presented as means and standard deviations. (**b**) *p* values resulted from pairwise comparisons between membranes’ thicknesses before immersion (initial thickness or thickness at t0: Th0) and after the different immersion periods. Statistical significance was set at *p* < 0.001.

(**a**)															
	**Biocollagen**			**Heart**			**Evolution X-Fine**			**CopiOs**			**Parasorb Resodont**		
	**Tryp** **.**	**CH**	**PBS**	**Tryp.**	**CH**	**PBS**	**Tryp.**	**CH**	**PBS**	**Tryp.**	**CH**	**PBS**	**Tryp.**	**CH**	**PBS**
t0	0.17 (0.01)	0.17 (0.01)	0.17 (0.01)	0.25 (0.07)	0.3 (0.17)	0.53 (0.11)	0.17 (0.06)	0.18 (0.05)	0.2 (0.05)	0.32 (0.09)	0.4 (0.06)	0.26 (0.07)	0.17 (0.01)	0.15 (0.01)	0.18 (0.01)
1 h	0.25 (0.06)	0.23 (0.02)	0.31 (0.16)	0.17 (0.08)	0.16 (0.08)	0.33 (0.09)	0.09 (0.04)	0.08 (0.02)	0.1 (0.03)	0.39 (0.11)	0.14 (0.03)	0.1 (0.01)	0.07 (0.02)	0.05 (0.01)	0.05 (0.01)
6 h	0.15 (0.05)	0.19 (0.04)	0.26 (0.11)	0.13 (0.02)	0.2 (0.07)	0.26 (0.11)	0.11 (0.04)	0.09 (0.03)	0.11 (0.04)	0.37 (0.06)	0.12 (0.02)	0.1 (0.01)	0.05 (0.02)	0.04 (0.01)	0.05 (0.01)
24 h	0.07 (0.05)	0.02 (0.03)	0.23 (0.12)	0.14 (0.04)	0.09 (0.03)	0.29 (0.04)	0.11 (0.05)	0.07 (0.02)	0.14 (0.06)	0.31 (0.11)	0.11 (0.02)	0.1 (0.01)	0.05 (0.02)	0.02 (0.03)	0.05 (0.02)
48 h	0.06 (0.04)	0 (0)	0.1 (0.06)	0.09 (0.03)	0.07 (0.02)	0.29 (0.06)	0.1 (0.05)	0.11 (0.02)	0.13 (0.06)	0.12 (0.02)	0.09 (0.02)	0.1 (0.01)	0.08 (0.02)	0 (0)	0.04 (0.01)
7 d	0.08 (0.06)	0 (0)	0.04 (0.06)	0.08 (0.03)	0 (0)	0.14 (0.03)	0.08 (0.04)	0.07 (0.06)	0,1 (0,04)	0.09 (0.02)	0 (0)	0.09 (0.02)	0.05 (0.03)	0 (0)	0.06 (0.01)
14 d	0.1 (0.1)	0 (0)	0.04 (0.06)	0.05 (0.01)	0 (0)	0.09 (0.02)	0.06 (0.03)	0.06 (0.08)	0.08 (0.03)	0.09 (0.03)	0 (0)	0.08 (0.01)	0.03 (0.03)	0 (0)	0.08 (0.01)
28 d	0.11 (0.08)	0 (0)	0.06 (0.09)	0.06 (0.02)	0 (0)	0.08 (0.01)	0.07 (0.02)	0.06 (0.09)	0.09 (0.04)	0.09 (0.04)	0 (0)	0.08 (0.02)	0.04 (0.03)	0 (0)	0.05 (0.02)
50 d	0.07 (0.07)	0 (0)	0.04 (0.06)	0.05 (0.01)	0 (0)	0.09 (0.02)	0.07 (0.05)	0.02 (0.03)	0.07 (0.02)	0.08 (0.02)	0 (0)	0.08 (0.02)	0.02 (0.03)	0 (0)	0.01 (0.02)
(**b**)															
0–1 h	**0.001**	**<0.001**	0.025	**<0.001**	0.002	**<0.001**	**<0.001**	**<0.001**	**<0.001**	0.002	**<0.001**	**<0.001**	**<0.001**	**<0.001**	**<0.001**
0–6 h	0.184	0.0719	0.027	**<0.001**	0.114	**<0.001**	**<0.001**	**<0.001**	**<0.001**	0.007	**<0.001**	**<0.001**	**<0.001**	**<0.001**	**<0.001**
0–24 h	**<0.001**	**<0.001**	0.191	**<0.001**	0.002	**<0.001**	**<0.001**	**<0.001**	**<0.001**	0.864	**<0.001**	**<0.001**	**<0.001**	**<0.001**	**<0.001**
0–48 h	**<0.001**	**<0.001**	0.009	**<0.001**	0.003	**<0.001**	**<0.001**	0.005	**<0.001**	**<0.001**	**<0.001**	**<0.001**	**<0.001**	**<0.001**	**<0.001**
0–7 d	**0.001**	**<0.001**	**<0.001**	**<0.001**	**0.001**	**<0.001**	**<0.001**	**<0.001**	**<0.001**	**<0.001**	**<0.001**	**<0.001**	**<0.001**	**<0.001**	**<0.001**
0–14 d	0.081	**<0.001**	**<0.001**	**<0.001**	**0.001**	**<0.001**	**<0.001**	**<0.001**	**<0.001**	**<0.001**	**<0.001**	**<0.001**	**<0.001**	**<0.001**	**<0.001**
0–28 d	0.036	**<0.001**	**0.011**	**<0.001**	**0.001**	**<0.001**	**<0.001**	**<0.001**	**<0.001**	**<0.001**	**<0.001**	**<0.001**	**<0.001**	**<0.001**	**<0.001**
0–50 d	**0.001**	**<0.001**	**<0.001**	**<0.001**	**0.001**	**<0.001**	**<0.001**	**<0.001**	**<0.001**	**<0.001**	**<0.001**	**<0.001**	**<0.001**	**<0.001**	**<0.001**

Tryp: trypsine; CH: *Clostridium histolyticum* collagenase; PBS: phosphate buffered saline.

**Table 3:** (**a**) Weight values (g) of the five membranes (Biocollagen, Heart, Evolution X-fine, CopiOs and Parasorb Resodont) after being submitted to PBS, trypsin and *C. histolyticum* for 1 h to 50 d. Values are presented as means and standard deviations. (**b**) Obtained *p* values after pairwise comparisons between membrane weights before immersion (initial weight or weight at t0: W0) and after the different immersion periods. Significance was considered at *p* < 0.001.

(**a**)															
	**Biocollagen**			**Heart**			**Evolution X-Fine**			**CopiOs**			**Parasorb Resodont**		
	**Tryp** **.**	**CH**	**PBS**	**Tryp.**	**CH**	**PBS**	**Tryp.**	**CH**	**PBS**	**Tryp.**	**CH**	**PBS**	**Tryp.**	**CH**	**PBS**
t0	12.27 (0.33)	11.95 (0.37)	11.63 (0.59)	8.91 (2.43)	8.54 (1.04)	10.83 (0.99)	7.21 (1.8)	8.34 (2.31)	7.9 (1.39)	12.95 (2.17)	13.94 (0.59)	11.55 (1.41)	3.77 (0.3)	3.96 (0.25)	3.82 (0.37)
1 h	11.66 (0.28)	12.43 (0.28)	12.17 (0.52)	8.75 (2.55)	9.1 (0.99)	11.55 (0.95)	7.13 (1.93)	8.01 (1.72)	9.32 (1.79)	12.63 (1.98)	14.22 (0.83)	11.87 (1.47)	3.83 (0.27)	4.29 (0.32)	4.24 (0.33)
6 h	8.10 (0.19)	3.94 (1.64)	11.79 (0.45)	9.07 (2.4)	8.33 (1.18)	11.5 (1.03)	7.21 (1.74)	7.41 (1.99)	9.11 (1.73)	12.41 (1.91)	12.18 (0.21)	11.77 (1.31)	2.98 (0.41)	2.95 (0.41)	4.07 (0.48)
24 h	2.78 (0.31)	0.25 (0.37)	10.59 (0.48)	8.68 (2.43)	7.05 (1.26)	11.16 (0.99)	6.94 (1.72)	6.75 (2.13)	8.86 (1.7)	11.13 (1.94)	10.78 (0.59)	11.41 (1.35)	2.29 (0.36)	0.31 (0.47)	3.71 (0.38)
48 h	2.6 (0.25)	0 (0)	5.87 (2.97)	8.16 (2.32)	4.36 (0.46)	10.88 (0.97)	6.9 (1.72)	5.91 (2.52)	8.82 (1.57)	8.02 (1.36)	8.23 (0.46)	11.42 (1.4)	1.41 (0.91)	0 (0)	3.66 (0.57)
7 d	1.29 (0.98)	0 (0)	0.73 (1.1)	6.9 (2.23)	0 (0)	9.5 (1.18)	6.06 (1.9)	3.21 (2.8)	8.55 (1.72)	5.76 (0.28)	0 (0)	10.75 (1.49)	0.49 (0.34)	0 (0)	2.52 (0.29)
14 d	0.92 (0.77)	0 (0)	0.36 (0.54)	5.45 (1.63)	0 (0)	7.17 (0.98)	5.49 (1.67)	1.08 (1.62)	7.78 (1.68)	5.38 (0.31)	0 (0)	9.88 (1.49)	0.32 (0.26)	0 (0)	0.71 (0.37)
28 d	0.76 (0.62)	0 (0)	0.15 (0.22)	4.69 (1.44)	0 (0)	5.75 (1.01)	5.39 (1.66)	1.08 (1.62)	7.27 (1.61)	5.29 (0.15)	0 (0)	9.59 (1.25)	0.22 (0.17)	0 (0)	0.36 (0.17)
50 d	0.61 (0.59)	0 (0)	0.15 (0.23)	4.43 (1.34)	0 (0)	5.48 (0.97)	5.14 (1.65)	1 (1.5)	6.97 (1.34)	4.93 (0.18)	0 (0)	9.03 (1.04)	0.05 (0.08)	0 (0)	0.32 (0.49)
(**b**)															
0–1 h	**<0.001**	0.002	**<0.001**	0.099	**<0.001**	**<0.001**	0.189	0.393	0.06	0.04	0.011	**<0.001**	0.228	**<0.001**	**<0.001**
0–6 h	**<0.001**	**<0.001**	0.146	0.047	0.062	**<0.001**	1	0.072	0.013	**0.001**	**<0.001**	0.002	**<0.001**	**<0.001**	0.003
0–24 h	**<0.001**	**<0.001**	**<0.001**	**<0.001**	**<0.001**	**<0.001**	**<0.001**	0.005	0.047	**<0.001**	**<0.001**	0.045	**<0.001**	**<0.001**	0.008
0–48 h	**<0.001**	**<0.001**	**0.001**	**<0.001**	**<0.001**	0.352	**<0.001**	**<0.001**	0.035	**<0.001**	**<0.001**	**<0.001**	**<0.001**	**<0.001**	0.054
0–7 d	**<0.001**	**<0.001**	**<0.001**	**<0.001**	**<0.001**	**<0.001**	**<0.001**	**<0.001**	0.161	**<0.001**	**<0.001**	**<0.001**	**<0.001**	**<0.001**	**<0.001**
0–14 d	**<0.001**	**<0.001**	**<0.001**	**<0.001**	**<0.001**	**<0.001**	**<0.001**	**<0.001**	0.788	**<0.001**	**<0.001**	**<0.001**	**<0.001**	**<0.001**	**<0.001**
0–28 d	**<0.001**	**<0.001**	**<0.001**	**<0.001**	**<0.001**	**<0.001**	**<0.001**	**<0.001**	0.12	**<0.001**	**<0.001**	**<0.001**	**<0.001**	**<0.001**	**<0.001**
0–50 d	**<0.001**	**<0.001**	**<0.001**	**<0.001**	**<0.001**	**<0.001**	**<0.001**	**<0.001**	0.024	**<0.001**	**<0.001**	**<0.001**	**<0.001**	**<0.001**	**<0.001**

Tryp: Trypsine; CH: *Clostridium histolyticum* collagenase; PBS: phosphate buffered saline.

## Data Availability

The data presented in this study are available on request from the corresponding author.
